# 국내 제2형 당뇨병 환자의 정서와 의료인과의 상호작용이 자기관리행위와 당화혈색소에 미치는 영향: 횡단적 조사 연구

**DOI:** 10.7586/jkbns.26.002

**Published:** 2026-05-27

**Authors:** Sunjoo Lee, Jieun Cha

**Affiliations:** 1 Department of Nursing, Catholic Sangji University, Andong, Korea; 1 가톨릭상지대학교 간호학과; 2 College of Nursing & Research Institute of Nursing Innovation, Kyungpook National University, Daegu, Korea; 2 경북대학교 간호대학·간호혁신연구소

**Keywords:** Type 2 diabetes mellitus, Emotion, Self-management, Glycated hemoglobin A, 제2형 당뇨병, 정서, 자기관리행위, 당화혈색소

## Abstract

**Purpose:**

This study examined the effects of emotional experiences and interactions with healthcare providers on self-management behaviors and glycemic control, as measured by hemoglobin A1c (HbA1c), in patients with type 2 diabetes mellitus.

**Methods:**

This cross-sectional study included 120 patients recruited from six local clinics between April 12 and July 15, 2021. Data were collected using the Scale of Positive and Negative Experience, the Client Satisfaction Tool, the Summary of Diabetes Self-Care Activities Questionnaire, and HbA1c measurements. Descriptive statistics, t-tests, analysis of variance, Pearson correlation coefficients, and hierarchical regression analyses were performed using SPSS version 31.0.

**Results:**

The mean age of the participants was 60.06 years, and the mean duration of diabetes was 7.79 years. Hierarchical regression analysis showed that interactions with healthcare providers and smoking status explained 11.0% of the variance in self-management behaviors. Negative emotion, sex, presence of diabetic complications, and smoking status explained 27.0% of the variance in HbA1c levels.

**Conclusion:**

Negative emotions and the quality of interactions with healthcare providers were significant factors associated with self-management behaviors and glycemic control in patients with type 2 diabetes mellitus. These findings highlight the need for early identification of high-risk patients and for comprehensive management strategies that incorporate psychological support and smoking cessation interventions. In addition, regular, individualized education and feedback, together with empathetic communication that acknowledges patients’ emotional experiences, may be important components of effective diabetes care.

## 서론

### 1. 연구의 필요성

전 세계적으로 제2형 당뇨병은 가장 빠르게 증가하는 만성질환 중 하나로, 2050년에는 성인 8명 중 1명이 당뇨병을 앓게 될 것으로 예측된다[1]. 우리나라 역시 고령화와 비만의 증가, 신체 활동 감소와 같은 생활양식 변화에 따라 당뇨병 유병률이 꾸준히 상승하고 있다[2]. 이러한 급증은 개인의 건강 악화뿐 아니라 국가 의료 체계와 환자 및 가족에게 상당한 부담을 안겨준다[1].

대한당뇨병학회 DM Fact Sheet 보고에 따르면, 30세 이상 성인의 74.7%가 유병 사실을 인지하였고, 치료율은 70.9%로 비교적 높았으나, hemoglobin A1c (HbA1c, 당화혈색소)를 6.5% 미만으로 조절하는 환자는 32.4%에 불과하여 3명 중 1명 정도만 적절한 혈당 관리를 하고 있었다[2]. 환자의 상당수가 행동 변화와 생활 습관 조절의 어려움을 겪고 있어 임상 현장에서 보다 체계적이고 다차원적인 관리전략이 필요한 실정이다. 당뇨병 환자의 혈당조절이 불충분한 경우 심혈관질환, 신장질환, 신경병증 등 합병증 발생 위험이 급격히 증가하므로[1,2] 식이, 운동, 약물 복용, 자가 혈당 측정 등 일상생활에서의 지속적인 자기관리행위가 필수적이다[3,4]. 하지만, 이러한 자기관리행위는 단순한 지식이나 의지만으로 유지하기 어려우며, 개인적·정서적·환경적 요인이 복합적으로 작용한다[3].

특히, 제2형 당뇨병은 완치가 불가능하고 평생 관리가 필요한 만성질환의 특성상 환자에게 심리적 부담을 초래하며, 대부분 불안과 우울, 스트레스와 같은 부정 정서를 경험하게 된다[5]. 이러한 정서적 부담은 지속적인 식이 조절, 규칙적 운동, 정기적인 병원 방문 등과 같은 자기관리 요구와 상호작용하여 자기관리행위를 감소시키고 결과적으로 혈당조절을 방해하는 요인으로 작용할 수 있다[6]. 반면 긍정 정서는 자기관리행위의 지속과 실천 의지를 강화하며 스트레스 상황에서도 자기효능감을 유지하고 문제 해결 중심적 대응을 가능하게 하여 장기적으로는 혈당조절에 유리하게 작용한다[7,8]. 이처럼 정서 반응은 치료 동기, 자기관리행동, 궁극적 치료 성과에 직접적인 영향을 미치는 요인이므로 효과적인 당뇨병 관리를 위해 환자의 정서 상태를 이해하는 것이 필수적이다.

한편, 당뇨병 치료에서 의료인과 환자 간의 긍정적 상호작용은 환자의 자기관리 이행과 건강 결과를 향상시키는 핵심 요인으로 평가된다[9]. 실제로 의료인과의 상호작용 만족도는 환자의 치료 순응도와 밀접하게 연관되며, 특히 환자의 의견을 존중하고 선호하는 치료 방식을 제공할 때 치료 효과가 향상되었다[9,10]. 또한 당뇨병 치료와 관련된 정확한 정보와 신뢰를 기반으로 한 의료인의 지지를 받는 경우 자기관리행위가 증진되어 HbA1c 수치를 낮추는 데 기여하였다[3]. 이와 같이 제2형 당뇨병 환자의 건강행위와 혈당조절은 단순한 의학적 치료를 넘어 환자의 심리적 상태와 의료인과의 상호작용에 의해 영향을 받을 것으로 예상된다.

Cox의 ‘상호작용적 건강행위 모델(Interaction Model of Client Health Behavior, IMCHB)’은 건강행위에 영향을 미치는 요인을 설명하는 이론으로 대상자 고유 특성과 대상자와 전문가 간의 상호작용을 주요 요소로 다룬다[11,12]. 대상자 고유 특성에는 배경 변인과 내적 동기, 인지적 평가, 정서적 반응이 포함되는데, 이 중 정서적 반응의 경우 당뇨병 환자가 경험하는 다양한 정서적 측면을 건강행위와 관련지어 살펴볼 수 있다. 대상자와 전문가 간의 상호작용 요소는 대상자의 건강행위에 직접적인 영향을 미치는 요인으로, 대상자와 건강 전문가 사이의 소통 과정을 의미하며 정서적 지지, 건강 정보, 의사 결정적 통제, 전문적·기술적 역량 총 4가지로 구성된다[11]. 즉, 전문가와의 상호작용을 통해 유발되는 동기와 신뢰는 대상자의 건강행위를 결정하고 긍정적인 건강상태를 촉진시킬 수 있다[10,11]. 기존에 IMCHB를 사용한 연구들은 의사소통과 같은 의료인과의 상호작용에 주로 초점을 맞추어 대상자 고유 특성이 충분히 고려되지 못하였고, 다양한 건강결과 중 대부분 주관적 지표 중심이라 객관적 임상 지표를 포괄적으로 다루지 못했다는 한계점이 있었다[12,13]. 본 연구에서는 이러한 제한점을 보완하여 제2형 당뇨병 환자의 정서적 반응과 의료인과의 상호작용을 함께 다루고 자기관리행위와 객관적 지표인 HbA1c에 미치는 영향을 분석하고자 한다.

종합하면, Cox의 상호작용적 건강행위 모델은 대상자 고유 특성과 대상자와 전문가 간의 상호작용을 통하여 건강 결과를 도출하는 통합적인 과정으로 제2형 당뇨병 환자의 자기관리행위와 궁극적인 치료 결과인 혈당조절과 관련된 다양하고 복합적인 심리사회적 변수를 규명하는데 적절한 이론적 기틀이 될 수 있다. 이에 본 연구에서는 Cox의 상호작용적 건강행위 모델의 일부 개념을 참고하여 제2형 당뇨병 환자의 정서와 의료인과의 상호작용이 자기관리행위와 당화혈색소에 미치는 영향을 확인하고자 한다.

### 2. 연구의 목적

본 연구의 목적은 제2형 당뇨병 환자의 정서와 의료인과의 상호작용이 자기관리행위와 당화혈색소에 미치는 영향을 규명하기 위함이며, 구체적인 목적은 다음과 같다.

1) 제2형 당뇨병 환자의 일반적 특성과 당뇨 관련 특성에 따른 자기관리행위와 당화혈색소의 차이를 파악한다.

2) 제2형 당뇨병 환자의 정서, 의료인과의 상호작용, 자기관리행위, 당화혈색소의 정도를 파악한다.

3) 제2형 당뇨병 환자의 정서, 의료인과의 상호작용, 자기관리행위와 당화혈색소 간의 상관관계를 파악한다.

4) 제2형 당뇨병 환자의 정서와 의료인과의 상호작용이 자기관리행위와 당화혈색소에 미치는 영향을 파악한다.

## 연구 방법

### 1. 연구 설계

본 연구는 제2형 당뇨병 환자의 정서와 의료인과의 상호작용이 자기관리행위와 당화혈색소에 미치는 영향을 파악하기 위한 서술적 조사 연구이다. 본 연구는 관찰연구 보고지침인 STROBE (Strengthening the Reporting of Observational Studies in Epidemiology) statement를 참고하여 연구 내용을 보고하였다.

### 2. 연구 대상

본 연구의 대상자는 경상북도 안동시에 거주하는 30세 이상의 제2형 당뇨병 환자로 Keum, Suh와 Han [6]의 연구를 참고하여 자기관리행위가 안정적으로 형성되기 위해서는 질병에 대한 이해와 생활습관 적응을 위한 기간이 필요하므로 당뇨 진단 후 6개월 이상이 경과하고 최근 3개월 이내에 HbA1c 검사를 받은 사람으로 그 결과에 대해 알고 있는 대상자를 임의 표집하였다. 반면, 제1형 또는 기타 유형의 당뇨병 환자, 진단 기간이 6개월 미만인 자, HbA1c 검사 결과를 확인할 수 없거나, 결과에 대한 인지가 불확실한 경우 연구에서 제외하였다.

표본 수는 G*Power 3.1 프로그램[14]을 이용하여 예측변수는 일반적 특성, 당뇨 관련 특성, 긍정 정서, 부정 정서, 의료인과의 상호작용 등 총 10개의 변수를 포함하였으며 중간 수준의 효과크기인 0.15, 유의수준(α) 0.05, 검정력(power) 0.80으로 설정하여 분석한 결과, 최소 요구 표본수는 118명으로 산출되었다. 약 10%의 탈락률을 고려하여 총 130부의 설문지를 배부하였고 최종적으로 회수된 설문지 120부를 본 연구의 자료 분석에 활용하였다.

### 3. 연구 도구

#### 1) 정서

Diener 등[15]이 개발한 긍정적 및 부정적 경험 척도(Scale of Positive and Negative Experience, SPANE)를 Koo [16]가 한국어로 타당화한 도구를 사용하였다. SPANE는 지난 4주간의 정서 경험 빈도를 평가하는 자기보고식 도구로, 총 12개의 정서 단어로 구성되어 있다. 각 문항은 1점(매우 드물게 또는 전혀 느끼지 못했다)에서 5점(거의 항상 느꼈다)까지의 5점 Likert 척도로 응답한다. 긍정 정서 6문항(‘긍정적인’, ‘좋다’, ‘유쾌한’, ‘기쁜’, ‘즐거운’, ‘만족한’)과 부정 정서 6문항(‘부정적인’, ‘나쁘다’, ‘불쾌한’, ‘슬픈’, ‘두려운’, ‘화난’)의 두 하위 요인으로 구성되며 각 하위척도의 점수 범위는 6점에서 30점이다. 점수가 높을수록 해당 정서의 경험 빈도가 높음을 의미한다. 긍정 정서에서 부정 정서를 뺀 정서균형 점수는 -24에서 24점까지 가능하다. Koo [16]의 연구에서 해당 도구는 긍정 정서 및 부정 정서 각각의 하위 요인에 대해 우수한 구성타당도를 보였으며, 신뢰도 Cronbach’s α는 Koo [16]의 연구에서 긍정 정서 .89, 부정 정서 .85였으며, 본 연구에서는 각각 .88과 .90이었다.

#### 2) 의료인과의 상호작용

Cox의 상호작용적 건강행위 모델에 기초하여 Bear와 Bowers [17]가 개발한 대상자 만족 도구(Client Satisfaction Tool)를 Choi [18]가 한국어로 번안한 버전을 사용하였다. 이 도구는 정서적 지지 2문항, 의사 결정적 통제 1문항, 건강정보 1문항, 전문적•기술적 능력 2문항, 의료인과 환자 간 관계에 대한 전반적인 만족도 1문항으로 총 7문항으로 구성된다. 각 문항은 5점 Likert 척도로 1점(전혀 동의하지 않는다)에서 5점(전적으로 동의한다)까지 응답하며, 총점은 7점에서 35점까지 가능하다. 점수가 높을수록 의료인과 대상자 간 관계의 질이 높음을 의미한다. Choi [18]의 연구에서 해당 도구는 내용타당도 및 구성타당도가 검증되었으며, 신뢰도 Cronbach’s α는 .86이었고 본 연구에서는 .91이었다.

#### 3) 자기관리행위

Toobert, Hampsom과 Glasgow [19]이 개발한 당뇨병 자가관리 활동 요약 척도(Summary of Diabetes Self-Care Activities Questionnaire, SDSCA)를 Chang과 Song [20]이 한국어로 타당화한 도구를 사용하였다. SDSCA는 총 17문항으로 구성되며 식이 관리 5문항, 운동 2문항, 약물복용 3문항, 혈당검사 2문항, 발 관리 5문항으로 세분화되어 있다. 각 문항은 ‘지난 7일 동안 해당 행위를 며칠 동안 했습니까?’라는 질문에 대해 응답자가 0일부터 7일까지의 숫자 중 해당 활동을 수행한 일수를 선택하도록 되어 있다. 점수가 높을수록 자기관리행위의 실천 정도가 높음을 의미하며, 가능한 총점 범위는 0점에서 119점이다. Chang과 Song [20]의 연구에서 해당 도구는 내용타당도와 구성타당도가 검증되었으며, 신뢰도 Cronbach’s α는 .77이었고 본 연구에서도 .77로 나타났다.

#### 4) 당화혈색소(HbA1c)

HbA1c는 적혈구 수명 동안 혈당과 결합된 혈색소의 비율을 나타내는 지표로 최근 약 3개월간의 평균 혈당 수준을 반영하므로 당뇨병의 진단과 치료 평가 시 중요하다. HbA1c의 측정 주기는 보통 2∼3개월이지만 대상자의 임상적 상황, 치료 방법 등을 반영해 혈당 변화가 심하거나 약물을 변경할 때, 또는 임신과 같이 철저한 관리가 필요한 경우 더 자주 검사할 수 있다. 본 연구에서 당화혈색소 검사 주기는 대상자가 다니는 병원의 임상의가 환자의 혈당조절 상태를 고려하여 결정하였다. 검사는 외래 진료 시 채혈한 검체를 전문 검사 기관에 의뢰하였고 의료기관으로부터 당화혈색소 수치를 통보받은 대상자가 설문지에 자가 보고 방식으로 작성하였다.

### 4. 자료수집 및 윤리적 고려

본 연구는 경북대학교 생명윤리심의위원회(IRB No. 2021-0046)의 승인을 받은 후, 2021년 4월 12일부터 7월 15일까지 자료를 수집하였다. 연구자는 내과 병원 4곳, 신경외과 병원 1곳, 외과 병원 1곳을 직접 방문하여 병원장 또는 외래 담당자에게 연구 목적과 절차를 상세히 설명하고 구두 동의를 받았다. 이후 ‘연구 참여자 모집 공고’를 부착하여 참여를 희망하는 대상자를 모집하였다. 참여 희망자에게는 연구 목적과 참여 방법을 대면으로 설명하고 자발적으로 동의한 경우 서면 동의서를 받은 후 설문지를 배부하였다. 설문지는 대면(126부)과 비대면(우편 또는 전자우편, 4부) 방식으로 배포하고 회수하였다. 연구자는 설문지 배부 시, 대상자에게 연구 목적과 설문지 작성(특히, HbA1c 수치를 최근 3개월 이내 결과로 기입)에 대해 구두로 상세히 설명하였고 대상자가 정확하게 이해하고 응답할 수 있도록 도왔다. 코로나19 감염 위험을 최소화하기 위해 질병관리청의 방역 지침을 철저히 준수하였으며, 조사 시 대상자와 연구자 간 물리적 거리를 유지할 수 있는 독립된 공간에서 진행하였다. 우편 회수 시 개인정보 보호를 위해 발신인 정보를 생략하고 수신자 주소만 기재하는 등 정보 유출 및 식별 위험을 최소화하였다. 본 연구는 참여자의 자율성과 비밀보장을 최우선으로 하였으며, 수집된 자료는 익명으로 처리하여 연구 목적 이외의 용도로 사용하지 않았다. 연구 참여는 언제든지 자유롭게 철회할 수 있음을 명확히 안내하였다.

### 5. 자료 분석

수집된 자료는 SPSS Statistics 31.0 (IBM Corp., Armonk, NY, USA) 프로그램을 이용하여 분석을 수행하였으며 구체적인 방법은 아래와 같다.

1) 대상자의 일반적 특성 및 당뇨 관련 특성은 기술통계로 분석하였다.

2) 대상자의 일반적 특성 및 질병 관련 특성에 따른 자기관리행위와 당화혈색소의 차이를 확인하기 위하여 t-test와 ANOVA로 분석하였고, 사후 검증은 Scheffé를 이용하였다

3) 대상자의 정서, 의료인과의 상호작용, 자기관리행위, 당화혈색소는 평균, 표준편차, 최솟값, 최댓값을 구하였다.

4) 대상자의 정서, 의료인과의 상호작용, 자기관리행위, 당화혈색소 간의 상관관계를 확인하기 위하여 Pearson correlation coefficient로 분석하였다.

5) 대상자의 정서와 의료인과의 상호작용이 자기관리행위와 당화혈색소에 미치는 영향을 확인하기 위해 위계적 회귀분석(hierarchical regression analysis)을 실시하였다.

## 연구 결과

### 1. 일반적 특성과 당뇨 관련 특성

대상자는 총 120명으로 남성이 53명(44.2%), 여성은 67명(55.8%)이었다. 평균 연령은 60.06 ± 12.92세였으며 50∼59세가 36명(30.0%)으로 가장 많았다. 교육 수준은 고등학교 이하가 77명(64.2%)이었으며 월수입은 300만원 이상인 대상자가 47명(39.2%)이었다. 가족과 동거하는 대상자는 100명(83.3%)이었으며, 이 중 56명(46.7%)은 가족으로부터 당뇨병 관리에 대한 도움을 받고 있었다.

당뇨 관련 특성으로 유병 기간은 5년 이하가 64명(53.4%)이었고, 평균 7.79 ± 7.07년이었다. 동반질환이 있는 대상자는 68명(56.7%), 당뇨병 관련 합병증이 있는 대상자는 19명(15.8%)이었다. 당뇨병으로 인한 입원 경험은 17명(14.2%)이 있었으며, 당뇨 교육을 받은 경험이 있는 대상자는 61명(50.8%)이었다. 음주는 67명(55.8%)이 ‘마신다’라고 응답하였고, 비흡연자는 98명(81.7%)이었다. 주관적 건강상태는 ‘보통’이라고 응답한 대상자가 55명(45.8%)으로 가장 많았다(Table 1).

### 2. 일반적 특성과 당뇨 관련 특성에 따른 자기관리행위와 당화혈색소의 차이

일반적 특성에 따른 자기관리행위 차이를 분석한 결과, 성별(t = −2.20, *p* = .030)에 따라 통계적으로 유의한 차이를 보였고 여성이 남성보다 자기관리행위 점수가 높았다. HbA1c의 차이를 분석한 결과, 성별(t = 2.12, *p* = .036)과 교육 수준에서 유의한 차이가 나타났다(t = 2.47, *p* = .015). 여성의 HbA1c가 남성보다 낮았으며, 교육 수준이 높은 집단에서 HbA1c가 낮았다(Table 1).

당뇨 관련 특성에 따른 자기관리행위 차이를 분석한 결과, 음주(t = -2.12, *p* = .036), 흡연(t = −3.41, *p* = .001)에서 통계적으로 유의한 차이를 보였다. 비음주자의 자기관리행위 점수가 음주자보다 유의하게 높았으며, 비흡연자의 자기관리행위 점수가 흡연자보다 유의하게 높았다. HbA1c의 차이를 분석한 결과, 유병 기간(F = 7.95, *p* = .001), 당뇨병 관련 합병증 여부(t = 3.64, *p* < .001), 당뇨병으로 인한 입원 경험(t = 2.59, *p* = .011), 흡연 여부(t = 2.87, *p* = .005), 주관적 건강상태(F = 5.78, *p* = .004)에 따라 통계적으로 유의한 차이가 있었다. 사후 검증 결과 당뇨 유병 기간이 5～10년 집단과 10년 초과 집단이 5년 이하 집단에 비해 HbA1c가 높았고, 당뇨 합병증이 있거나 입원 경험이 있는 경우, 그리고 흡연자가 유의하게 높았으며, 주관적 건강상태는 ‘불건강하다’라고 대답한 집단이 ‘건강하다’와 ‘보통이다’고 응답한 집단보다 당화혈색소가 높았다(Table 1).

### 3. 대상자의 정서, 의료인과의 상호작용, 자기관리행위, 당화혈색소 정도

대상자의 정서 수준을 살펴본 결과, 긍정 정서는 19.11 ± 4.06점, 문항당 3.19 ± 0.68점, 부정 정서는 14.46 ± 4.84점, 문항당 2.41 ± 0.99점, 정서 균형은 평균 4.63 ± 7.63점이었고 최소 −12점, 최대 22점으로 나타났다. 의료인과의 상호작용 점수는 23.93 ± 4.69점 문항당 3.42 ± 0.84점, 자기관리행위는 59.45 ± 16.44점, 문항당 3.50 ± 0.97점이었다. HbA1c 수치의 평균은 6.71 ± 0.64%이었고, 최소 5.40%, 최대 9.00%로 나타났다(Table 2).

### 4. 대상자의 정서, 의료인과의 상호작용, 자기관리행위, 당화혈색소 간의 상관관계

대상자의 자기관리행위는 긍정 정서, 부정 정서, 정서 균형, 의료인과의 상호작용과 유의한 상관관계가 나타나지 않았다. HbA1c는 부정 정서(r = .27, *p* = .003)와 정적 상관관계, 정서 균형(r = −.25, *p* = .007) 및 자기관리행위(r = −.35, *p* < .001)와 부적 상관관계가 나타났다(Table 3).

### 5. 대상자의 정서, 의료인과의 상호작용이 자기관리행위에 미치는 영향

먼저 회귀분석의 적절성을 검토하기 위하여 독립변수(긍정 정서, 부정 정서, 의료인과의 상호작용)와 종속변수(자기관리행위) 간의 선형성 가정을 확인하였고 표준화 예측값과 표준화 잔차의 산점도에서 잔차가 0을 중심으로 무작위하게 분포하여 선형성 가정을 충족하였다. 잔차의 독립성을 검토하기 위해 Durbin-Watson 값을 확인하였고 1.66으로 2에 가까워 잔차 간 자기상관이 없었다. 잔차의 정규성은 표준화 잔차의 히스토그램을 통해 확인하였고 정규분포 곡선에 근접한 분포를 보여 충족하였다. 잔차의 등분산성은 표준화 예측값과 표준화 잔차의 산점도를 통해 검토하였고 잔차가 표준화 예측값의 전 범위에 걸쳐 0을 중심으로 균등하게 분포하여 등분산성 가정을 충족하였다. 다중공선성 검토 결과, 공차한계(tolerance)는 .30～.87로 기준치 0.1 이상이었으며, 분산팽창지수는 1.15～3.30으로 기준치 10 미만으로 다중공선성은 확인되지 않았다.

덧붙여 위계적 회귀분석은 연구모형에 근거하여 변수들을 단계적으로 투입함으로써 각 변수군의 추가 설명력을 검증하는 방법이며[21], 본 연구는 IMCHB를 이론적 틀로 적용하였으므로 단순 상관관계의 유의성 여부와 관계없이 긍정 정서, 부정 정서, 의료인과의 상호작용을 핵심 개념 변수에 포함하였다. 단, 정서 균형의 경우 긍정 정서와 부정 정서의 조합으로 계산되는 지표이므로, 동시에 회귀모형에 포함될 경우 개념적 중복 및 다중공선성 문제가 발생할 수 있어 제외하였다.

자기관리행위에 영향을 미치는 요인을 확인하기 위해, 성별, 음주, 흡연을 더미변수로 하여 투입한 1단계 분석(Model 1)을 먼저 실시하였다. 그 결과 모형은 통계적으로 유의하였으며(F = 4.55, *p* = .005), 설명력은 8.0%였다. 다음으로 긍정 정서, 부정 정서 및 의료인과의 상호작용을 추가한 2단계 분석(Model 2)에서도 역시 통계적으로 유의하였고(F = 3.51, *p* = .003), 모형의 설명력은 11.0%로 증가하였다(Table 4). 흡연은 여전히 유의한 예측 변수였으며(β = .24, *p* = .019), 의료인과의 상호작용도 자기관리행위의 유의한 예측 변수로 나타났다(β = .19, *p* = .041). 반면 긍정 정서, 부정 정서는 자기관리행위의 유의한 예측 변수가 아니었다. 제2형 당뇨병 환자 중 비흡연자가 자기관리행위가 높고, 의료인과의 상호작용이 높을수록 자기관리행위가 높아짐을 의미하였다.

### 6. 대상자의 정서, 의료인과의 상호작용이 당화혈색소에 미치는 영향

회귀분석의 조건을 확인하고자 독립변수(긍정 정서, 부정 정서, 의료인과의 상호작용)와 종속변수(HbA1c) 간의 선형성 가정을 검토하였고 표준화 예측값과 표준화 잔차의 산점도에서 잔차가 0을 중심으로 무작위하게 분포하였다. Durbin-Watson 값은 1.72로 잔차 간 자기상관이 없었으며, 잔차의 정규성은 표준화 잔차의 히스토그램을 통해 확인하였고 정규분포 곡선에 근접한 분포를 보여 충족하였다. 잔차의 등분산성은 표준화 예측값과 표준화 잔차의 산점도를 통해 검토한 결과, 잔차가 표준화 예측값의 전 범위에 걸쳐 0을 중심으로 균등하게 분포하여 등분산성 가정을 충족하였다. 다중공선성 검토 결과, 공차한계는 .57 ~ .92, 분산팽창지수는 1.09 ~ 1.75로 문제가 없었다.

HbA1c에 영향을 미치는 요인을 확인하기 위해, 성별, 교육 수준, 유병 기간, 당뇨병 관련 합병증 여부, 당뇨병 관련 입원 경험, 흡연, 주관적 건강상태를 더미변수로 하여 투입한 1단계 분석을 먼저 실시하였다. 그 결과, 모형은 통계적으로 유의하였으며(F = 5.72, *p* < .001), 설명력은 26.0%였다(Adj. R^2^ = .26). 긍정 정서, 부정 정서 및 의료인과의 상호작용을 추가한 2단계 분석에서도 모형은 통계적으로 유의하였으며(F = 4.72, *p* < .001), 설명력은 다소 증가하여 27.0%였다. 성별(β = −.25, *p* = .010), 당뇨병 관련 합병증(β = −.20, *p* = .033), 흡연(β = −.21, *p* = .030)과 함께 부정 정서(β = .20, *p* = .038)는 HbA1c를 유의하게 증가시키는 요인으로 확인되었다. 반면, 긍정 정서와 의료인과의 상호작용은 HbA1c에 유의하지 않았다. 제2형 당뇨병 환자 중 여성, 당뇨병 관련 합병증이 없는 경우, 비흡연자가 HbA1c 수치가 낮고, 부정 정서가 높을수록 HbA1c가 높아짐을 의미하였다(Table 5).

## 논의

본 연구는 지속적인 치료와 혈당조절이 요구되는 만성질환인 제2형 당뇨병 환자를 대상으로 Cox의 상호작용적 건강행위 모델을 참고하여 정서와 의료인과의 상호작용이 자기관리행위와 당화혈색소에 미치는 영향을 분석하고자 수행되었다. 특히 Cox 모델은 대상자의 개별성과 대상자와 의료인의 관계, 그리고 이로 인한 건강 결과를 구체화하므로 전인적 접근과 치료적 관계를 중요시하는 간호 실무를 설명하는 프레임워크로 적절하다고 보았다.

대상자의 정서를 먼저 살펴보면 긍정 정서가 19.11점, 부정 정서가 14.46점으로 당뇨라는 만성적인 스트레스 상황에서도 긍정 정서가 부정 정서보다 높게 나타나 질병 대처 과정에서 힘든 부분이 있더라도 긍정적으로 적응해가고 있음을 확인하였다. 기존에 주로 다루던 우울, 불안과 같은 부정적 정서 외에도 앞으로 보다 균형적인 접근을 위해 만족감과 기쁨 등의 다양한 긍정 정서도 함께 고려하는 것이 필요하다고 하겠다[22].

대상자와 의료인과의 상호작용은 100점으로 환산 시 68.4점으로 대체로 만족하는 편이었다. 하위 영역별로 살펴보면 의료진과의 관계 만족도 3.53점, 건강 정보 3.50점, 정서적 지지 3.45점, 의사 결정적 통제 3.37점, 전문적·기술적 능력 3.32점의 순이었다. 점수가 가장 낮았던 전문적·기술적 능력은 ‘의료진들은 나의 이해력 정도에 맞게 내 질문에 대한 답을 준다’, ‘내가 받고 있는 치료와 간호의 질은 높다’의 2개 항목으로 구성되었는데, 이는 일차 의료기관의 진료 특성상 치료 중심으로 진행되며, 의료진과 환자가 대면하는 시간이 매우 짧아 환자의 개별적 여건이 충분히 고려되지 않는 진료 시스템이 반영된 것으로 보인다.

자기관리행위는 100점으로 환산 시 49.6점으로 절반에 못 미치는 수준으로 하위영역별로 살펴보면 투약이 가장 높았고 식이요법, 발 관리, 운동, 혈당 검사의 순이었다. 동일한 도구(SDSCA)를 사용하였던 Keum과 Suh [23]의 65점, Oh와 Lee [3]의 67점과 비교 시 낮은 편이었다. 이러한 차이는 선행 연구의 대상자가 도시지역 및 종합병원 이용자였던 반면 본 연구는 농촌 지역 거주자 및 일차 의료기관 이용자라는 점에서 지역과 의료기관에 따른 의료접근성의 차이가 반영된 것으로 사료된다.

HbA1c의 평균은 6.71%로 권장 기준인 6.5% 미만으로 유지되지 못했고, 6.5% 미만으로 유지하고 있는 대상자는 38.3%를 차지하여 과반수 이상에서 혈당 관리가 제대로 이루어지지 않았다. Jeon 등[24]이 성인 당뇨병 환자를 대상으로 조사했을 때 HbA1c는 6.88%였고, 6.5% 미만의 조절군은 21.9%였던 반면, Lee [25]가 노인 당뇨병 환자에서 보고한 HbA1c는 7.12%였고, 6.5% 미만의 조절군은 38.5%로 연구마다 상이하였다. 이러한 차이는 연구 대상의 연령, 당뇨병 이환 기간, 자기관리 수준 등의 차이에서 기인했을 것으로 보인다. 본 연구에서는 50~60대 중년층이 많았으나 가족으로부터 돌봄 제공을 받는 대상자가 절반 이하에 그쳐 혈당관리의 어려움이 있었을 것으로 사료된다.

일반적 및 당뇨 관련 특성 중 성별과 흡연의 경우 자기관리행위와 HbA1c에 모두 유의한 관련성을 보였다. 남성의 경우 직장 생활과 외부 활동으로 인한 불규칙한 식사, 운동 부족 등이 발생하기 쉬우므로 개인별 맞춤형 자기관리 전략이 요구된다. 흡연은 인슐린 저항성을 높여 당화혈색소를 증가시키며[26], 흡연자가 의료인과의 관계에서도 소극적이며 치료 불이행률이 높은 것으로 알려졌다[27]. 따라서 당뇨병 교육 시 흡연과 혈당조절 간의 생리적 연관성을 강조하고, 필요시 금연 치료 프로그램을 적극 연계해야 할 것이다.

위계적 회귀분석 결과, 본 연구에서 제2형 당뇨병 환자의 자기관리행위 영향 요인은 흡연과 의료인과의 상호작용이었으며 이들 변인이 11.0%를 설명하였다. 이 결과는 흡연 상태와 의료인과의 상호작용이 당뇨 환자의 자기관리 행동 수행에 중요한 요인임을 시사하며, 의료인과의 적극적인 상호작용을 통해 자기관리 행동을 강화할 필요가 있음을 보여준다. 의료인과의 상호작용은 다양한 변수들과 동시에 영향을 주고 받으므로 단변량 분석에서는 자기관리행위와 유의한 상관관계가 없었으나 다른 변수들의 영향을 통제한 상태에서 각 변수의 고유한 설명력을 평가하는 다변량 분석에서는 유의미한 영향력을 나타냈다. 따라서 Cox의 상호작용적 건강행위 모델 중 대상자와 전문가 간의 상호작용이 건강행위에 영향을 준다는 것을 지지하였다[11,12]. 즉, 의사와 간호사가 환자의 정서적 어려움이나 생활 습관 문제를 이해하고 지속적으로 지지와 피드백을 제공한다면 자기관리 수행 수준이 높아질 수 있다. 이는 선행 연구들과도 일치하며[9], 임상 현장에서 시간적 제약과 진료의 한계가 있겠지만, 당뇨병 관리에서 의료인의 관계적 접근과 교육적 지원을 보다 강화해야 할 것이다.

HbA1c에 영향을 미치는 요인은 성별, 이환 기간, 당뇨 관련 합병증, 흡연, 부정 정서로 나타났고 변수들의 설명력은 27.0%였다. 특히 부정 정서가 혈당 조절에 유의한 영향을 준 점은 주목할 필요가 있겠다. 부정 정서는 스트레스 호르몬 분비를 증가시켜 인슐린 감수성을 낮추는[28] 한편, 치료 동기를 저하시키고 의료진과의 관계에서 비협력적 관계를 촉진하므로 당뇨병 치료에 있어 부정적인 것으로 보고되었다[24,29]. 또한 부정적인 감정은 면역력 저하와 염증반응 등과도 관련 있어 당뇨병과 같은 만성질환의 치유를 지연시킨다[8]. 부정 정서가 HbA1c에 유의한 설명력을 보인 것은 제2형 당뇨병이 장기간 관리가 요구되는 만성질환이라는 특성과도 관련 있다. 질병 관리 과정에서 반복적으로 경험하는 실패감, 부담감, 통제감의 상실은 시간이 지남에 따라 누적되어 부정 정서를 강화할 수 있으며, 이러한 정서 상태는 혈당 관리의 지속성을 저해하는 요인으로 작용할 가능성이 크다[5,8]. 따라서 당뇨병 환자의 정서는 단순한 심리적 반응이 아니라, 혈당 조절의 중요한 임상적 지표로 인식될 필요가 있다.

이 밖에 성별에 따라 혈당조절 경향이 상이할 수 있으며, 합병증을 보유한 환자의 경우 보다 엄격한 혈당 관리가 요구되었다. 본 연구에서 당뇨병 이환 기간이 5~10년인 군과 10년 초과 군이 5년 이하 군보다 HbA1c가 높았는데, 이는 질병이 장기화될수록 췌장의 *β*세포 기능이 저하되고, 인슐린 저항성이 증가하여 혈당조절이 어려워진다는 기존 연구[30]를 뒷받침하였다. 한편, 본 연구모형의 설명력은 높지 않았는데, 이는 인간의 건강행동과 혈당조절이 다양한 생물학적 및 환경적 요인의 영향을 받는 복합적 현상인 것과 관련이 있다. 따라서 향후 연구에서는 보다 다양한 개인적•환경적 요인을 포함한 모형 검증이 필요하겠다.

본 연구는 Cox의 상호작용적 건강행위 모델의 주요 개념 중 정서적, 관계적 요인에 초점을 두고 자기관리행위와 객관적 임상 지표인 HbA1c와의 관련성을 분석함으로써 생리적·정서적·행동적 요인을 통합하여 설명하였다. 의료인과 환자 간 상호작용의 질이 제2형 당뇨병 환자의 자기관리행위를 향상시키는 중요한 요인임을 확인함으로써, 임상 현장에서 지지적 의사소통, 지속적 피드백, 관계 형성 중심의 간호 중재가 강화되어야 함을 보여주었다. 특히 부정 정서가 HbA1c에 유의한 영향을 미친 결과는 정서 상태가 단순한 심리적 경험을 넘어 대사 항상성(metabolic homeostasis)에 직접적으로 영향을 미칠 수 있음을 시사한다. 즉, 부정 정서는 시상하부-뇌하수체-부신축과 교감신경계를 활성화하여 스트레스 호르몬 분비와 염증 반응을 증가시키고 인슐린 감수성을 저하시킴으로써 혈당조절 악화와 HbA1c 상승에 영향을 준다[28]. 따라서 간호사는 생리적 수치의 관리뿐 아니라 대상자의 정서 상태와 관계적 측면을 다루는 전문직으로서, 생리·심리·사회 영역을 통합적으로 중재하는 역할을 수행해야 할 것이다.

본 연구의 제한점은 일개 농촌 지역 의원 6곳에서 실시되었으며, 대상자 수가 적어 연구 결과를 제2형 당뇨병 환자 전체로 일반화하는데 주의가 필요하다. 또한 횡단적 자료이므로 변수 간의 인과관계를 살펴보기 위해서는 종단적 연구가 이루어져야 할 것이다. 특히, 약 5년 전에 수집된 자료이므로 최근의 임상 지침 변화, 치료 환경의 발전, 간호 실무 및 정책 변화 등 최신 의료환경을 충분히 반영하지 못했을 가능성이 있다. 따라서 향후 연구에서는 최근 자료를 활용하여 이러한 변화를 반영한 추가 검증이 필요하겠다. 덧붙여 당뇨병 연구에서는 행동, 심리 상태, 그리고 생리적 결과가 서로 다른 시간 범위를 통해 나타나는 특성을 고려하여 함께 분석하는 것이 일반적이지만[19], 본 연구에서 주요 변수들의 측정 기간이 서로 다른 시간 범위를 반영한다는 점을 유의해야 한다. 정서는 최근 4주, 자기관리행위는 최근 7일, 그리고 HbA1c는 3개월 동안의 평균 혈당 상태를 반영하는 지표이므로 변수 간 시간적 불일치가 결과 해석에 영향을 줄 수 있다. 따라서 향후 연구에서는 동일한 시간 범위를 반영하는 반복 측정이나 종단 연구를 통해 변수 간 관계를 보다 명확하게 규명할 필요가 있다. 본 연구 결과는 제2형 당뇨병 환자의 자기관리행위와 혈당조절을 향상시키기 위해 의료인과 대상자 간의 상호 관계의 질을 높이고, 대상자의 정서적 반응을 고려한 심리사회적 중재 개발에 기초자료로 활용할 수 있을 것으로 기대한다.

## 결론

본 연구는 제2형 당뇨병 환자의 정서, 의료인과의 상호작용, 자기관리행위, HbA1c의 정도를 확인하고 이들 간의 연관성 및 자기관리행위와 HbA1c에 영향을 미치는 요인을 규명하고자 실시하였다. 연구 결과 자기관리행위에 유의한 영향 요인은 의료인과의 상호작용이었으며, HbA1c에 영향을 미치는 요인은 성별, 당뇨 관련 합병증, 부정 정서이며 공통적 요인은 흡연으로 나타났다. 남성, 합병증 보유자, 흡연자, 그리고 부정 정서 수준이 높은 환자가 혈당조절 실패 위험이 높은 집단임을 확인하였다. 이에 따라 임상에서는 이들 고위험군을 조기에 선별하여 심리적 중재와 금연 프로그램을 포함한 다차원적 관리전략을 시행해야 할 필요가 있겠다. 이 밖에도 제2형 당뇨병 환자의 자기관리행위를 향상시키기 위해 의료인은 단순히 교육 내용을 전달하는 수준을 넘어 환자와의 상호작용을 적극적으로 강화할 필요가 있다. 환자의 이야기를 충분히 경청하고 공감하는 정서적 지지와 함께 환자 수준에 맞춘 교육과 피드백을 정기적으로 제공하여야 할 것이다.

본 연구 결과를 토대로 다음과 같이 제언하고자 한다. 첫째, 제2형 당뇨병 환자의 정서 관리와 의료인과의 상호작용 강화를 위한 간호 중재 개발 및 효과를 평가하는 추가 연구가 필요하다. 둘째, 본 연구는 일개 농촌 지역 의원 6곳에서 실시되었으므로 향후 다양한 지역과 더 많은 표본을 기반으로 한 반복 연구가 필요하다.

## Figures and Tables

**Table 1.** Differences in Self-management Behavior and HbA1c According to General and Diabetes-related Characteristics (N = 120)

Characteristics	Categories	n (%)	Self-management behaviors			HbA1c		
			M ± SD	t / F	*p* Scheffé	M ± SD	t / F	*p* Scheffé
Sex	Men	53 (44.2)	55.79 ± 17.04	−2.20	.030	6.84 ± 0.70	2.12	.036
	Women	67 (55.8)	62.34 ± 15.48			6.60 ± 0.57		
Age (years)	≤ 49	23 (19.2)	56.78 ± 18.64	1.68	.174	6.57 ± 0.55	2.09	.105
	50∼59	36 (30.0)	61.06 ± 14.24			6.77 ± 0.71		
	60∼69	28 (23.3)	54.93 ± 11.05			6.91 ± 0.56		
	≥ 70	33 (27.5)	63.39 ± 20.00			6.56 ± 0.65		
Education	≤ High school	77 (64.2)	59.08 ± 15.71	−0.33	.742	6.81 ± 0.65	2.47	.015
	≥ College	43 (35.8)	60.12 ± 17.86			6.52 ± 0.60		
Monthly Income (10,000 KRW)	< 200	40 (33.3)	59.58 ± 18.29	0.00	.997	6.64 ± 0.57	2.57	.081
	200∼300	33 (27.5)	59.52 ± 13.46			6.92 ± 0.59		
	≥ 300	47 (39.2)	59.30 ± 17.03			6.61 ± 0.71		
Cohabitation	Alone	20 (16.7)	54.35 ± 16.64	−1.53	.129	6.69 ± 0.55	−0.16	.874
	With family	100 (83.3)	60.47 ± 16.30			6.71 ± 0.66		
Family caregiver	Yes	56 (46.7)	57.27 ± 16.75	1.37	.175	6.60 ± 0.59	1.67	.097
	No	64 (53.3)	61.36 ± 16.06			6.80 ± 0.68		
Duration of disease (years)	≤ 5^a^	64 (53.4)	60.81 ± 17.82	0.47	.628	6.50 ± 0.56	7.95	.001
	6∼10^b^	28 (23.3)	57.89 ± 11.77			6.91 ± 0.52		a < b,c
	＞10^c^	28 (23.3)	57.89 ± 17.39			6.97 ± 0.77		
Comorbidities	Yes	68 (56.7)	58.31 ± 16.91	0.87	.387	6.73 ± 0.72	−0.46	.647
	No	52 (43.3)	60.94 ± 15.86			6.68 ± 0.53		
Diabetic complications	Yes	19 (15.8)	54.16 ± 12.95	−1.54	.127	7.17 ± 0.52	3.64	< .001
	No	101 (84.2)	60.45 ± 16.89			6.62 ± 0.63		
Admission due to diabetes	Yes	17 (14.2)	55.18 ± 16.91	−1.16	.249	7.07 ± 0.54	2.59	.011
	No	103 (85.8)	60.16 ± 16.34			6.65 ± 0.64		
Diabetes education	Yes	61 (50.8)	62.02 ± 17.37	1.75	.082	6.75 ± 0.62	0.75	.454
	No	59 (49.2)	56.80 ± 15.12			6.66 ± 0.67		
Drinking	Yes	67 (55.8)	56.66 ± 14.64	−2.12	.036	6.79 ± 0.69	1.53	.129
	No	53 (44.2)	62.98 ± 18.00			6.61 ± 0.57		
Smoking	Yes	22 (18.3)	49.09 ± 12.46	−3.41	.001	7.05 ± 0.80	2.87	.005
	No	98 (81.7)	61.78 ± 16.38			6.63 ± 0.58		
Subjective health status	Healthy^a^	23 (19.2)	57.48 ± 16.77	1.38	.256	6.51 ± 0.57	5.78	.004
	Normal^b^	55 (45.8)	62.15 ± 17.00			6.59 ± 0.62		a,b < c
	Unhealthy^c^	42 (35.0)	57.00 ± 15.37			6.96 ± 0.64		

M = Mean; SD = Standard deviation; HbA1c = Hemoglobin A1c; KRW = Korean Won.

**Table 2.** Emotions, Interactions with Healthcare Providers, Self-management Behaviors, and HbA1c (N = 120)

Variables	Categories	M ± SD	Min	Max	Item
					M ± SD
Emotions	Positive	19.11 ± 4.06	6.00	28.00	3.19 ± 0.68
	Negative	14.46 ± 4.84	6.00	25.00	2.41 ± 0.99
	Balance	4.63 ± 7.63	−12.00	22.00	
Interactions with healthcare providers		23.93 ± 4.69	15.00	35.00	3.42 ± 0.84
Self-management behaviors		59.45 ± 16.44	31.00	107.00	3.50 ± 0.97
HbA1c		6.71 ± 0.64	5.40	9.00	

M = Mean; SD = Standard deviation; Min = Minimum; Max = Maximum; HbA1c = Hemoglobin A1c.

**Table 3.** Correlations among Emotions, Interaction with Healthcare Providers, Self-management Behaviors, and HbA1c (N = 120)

	1	2	3	4	5	6
	r (*p*)					
1. Positive emotions	1	-	-	-	-	-
2. Negative emotions	−.47 (< .001)	1	-	-	-	-
3. Emotional balance	.83 (< .001)	−.88 (< .001)	1	-	-	-
4. Interaction with healthcare providers	.24 (.008)	−.00 (.977)	.13 (.157)	1	-	-
5. Self-management behaviors	.11 (.225)	−.08 (.368)	.11 (.224)	.18 (.053)	1	-
6. HbA1c	−.14 (.128)	.27 (.003)	−.25 (.007)	−.08 (.404)	−.35 (< .001)	1

HbA1c = Hemoglobin A1c.

**Table 4.** Factors Influencing Self-management Behaviors in Patients with Type 2 Diabetes Mellitus (N = 120)

Variables	Model 1				Model 2			
	B	SE	β	t (*p*)	B	SE	β	t (*p*)
Sex (women) (reference = men)	1.08	3.45	.03	0.31 (.754)	2.06	3.47	.06	0.59 (.555)
Drinking (no)^†^	3.86	3.11	.12	1.24 (.216)	4.69	3.14	.14	1.49 (.138)
Smoking (no)^‡^	10.78	4.28	.26	2.52 (.013)	10.11	4.24	.24	2.38 (.019)
Positive emotions	-	-	-	-	.28	.43	.07	0.66 (.511)
Negative emotions	-	-	-	-	-.21	.34	−.06	−0.60 (.550)
Interactions with healthcare providers	-	-	-	-	.65	.32	.19	2.07 (.041)
	R^2^ = .11, Adj. R^2^ = .08, F = 4.55, *p* = .005				R^2^ = .16, Adj. R^2^ = .11, F = 3.51, *p* = .003			

SE = Standard error.

Dummy variable: ^†^Drinking (reference = yes); ^‡^Smoking (reference = yes).

**Table 5.** Factors Influencing HbA1c in Patients with Type 2 Diabetes Mellitus (N = 120)

Variables	Model 1				Model 2			
	B	SE	β	t (*p)*	B	SE	β	t (*p)*
Sex (women) (reference = men)	−.29	.12	−.22	−2.37 (.020)	−.32	.12	−.25	−2.62 (.010)
Education (High school)^†^	−.21	.13	−.16	−1.69 (.094)	−.18	.13	−.13	−1.37 (.174)
Duration of disease^‡^ (years)								
6∼10	.27	.14	.18	1.97 (.051)	.27	.14	.18	1.97 (.051)
>10	.23	.15	.15	1.55 (.124)	.25	.15	.17	1.71 (.090)
Diabetic complications (yes) (reference = no)	−.36	.16	−.21	−2.24 (.027)	−.35	.16	−.20	−2.17 (.033)
Admission due to diabetes^§^	−.10	.17	−.05	−0.57 (.568)	−.10	.17	−.05	−0.59 (.556)
Smoking^∥^	−.38	.15	−.23	−2.47 (.015)	−.34	.15	−.21	−2.20 (.030)
Subjective health status^¶^								
Normal	.02	.14	.02	0.17 (.865)	.04	.15	.03	0.24 (.814)
Unhealthy	.19	.17	.14	1.11 (.268)	.15	.19	.11	0.80 (.424)
Positive emotions					.02	.02	.11	1.02 (.311)
Negative emotions					.03	.01	.20	2.10 (.038)
Interactions with healthcare providers					−.01	.01	−.05	−0.59 (.557)
	R^2^ = .32, Adj. R^2^ = .26, F = 5.72, *p* < .001				R^2^ = .35, Adj. R^2^ = .27, F = 4.72, *p* < .001			

HbA1c = Hemoglobin A1c; SE = Standard error. Dummy variable:

† Education (reference = ≥ college);

‡ Duration of disease (reference = ≤ 5 years);

§ Admission due to diabetes (reference = yes);

∥ Smoking (reference = yes);

¶ Health status (reference = healthy).
